# Hemophagocytic Lymphohistiocytosis and the Liver Masquerade

**DOI:** 10.7759/cureus.87133

**Published:** 2025-07-01

**Authors:** Hadasa Banayan, Jieqi Liu, Dominique Haoson

**Affiliations:** 1 Internal Medicine, Jersey City Medical Center/RWJ Barnabas Health, Jersey City, USA; 2 Hematology and Oncology, Division of Blood Disorders, Rutgers Cancer Institute of New Jersey, New Brunswick, USA

**Keywords:** acute alcoholic hepatitis, acute liver failure (alf), acute-on-chronic liver failure, alcoholic liver failure, chronic liver disease (cld), diffuse large b cell lymphoma (dlbcl), hemophagocytic lymphohistiocytosis (hlh), hereditary hemochromatosis (hh), hyperferritinemia, pancytopenia

## Abstract

We present a case of hemophagocytic lymphohistiocytosis (HLH) with a complex presentation that could have been easily mistaken as one of many alternative diagnoses. Our patient, with chronic alcohol use disorder, presented with new-onset liver symptoms and pancytopenia. This case underscores the importance of clinical suspicion and further investigation of a wide differential. The purpose of this case report is to highlight the diagnostic challenges and nuances in patient presentation that can mislead healthcare providers while emphasizing the ambiguity of liver failure symptoms in patients with undiagnosed HLH.

## Introduction

Hemophagocytic lymphohistiocytosis (HLH) is a rare and life-threatening syndrome of immune dysregulation driven by the failure of natural killer (NK) cells and cytotoxic lymphocytes to eliminate activated macrophages. This imbalance in normal feedback results in widespread inflammation, causing hypercytokinemia, tissue accumulation of activated macrophages, and end-organ damage caused by T-cells. HLH can be familial or spontaneous in origin, with primary or familial HLH mainly affecting pediatric patients less than 18 months of age [1]. Secondary or spontaneous HLH is more observed in adults and can arise in the context of infection, autoimmune and rheumatological disorders (macrophage activation syndrome), and hematological and solid organ malignancy and cancer treatment. Overall, HLH is known to have a high mortality rate up to 40% [2]. Furthermore, untreated malignancy-associated HLH (MAH) is known to have a median survival time as low as 2-6 months, making prompt diagnosis crucial for survival [3]. MAH accounts for 45% of adult HLH and a smaller proportion of pediatric HLH [4]. Lymphoma is the primary malignancy associated with MAH, with T/NK-cell lymphoma (35%), B-cell lymphoma (32%), and Hodgkin's lymphoma (6%) being the most common [5]. Lymphoma-associated HLH is further divided by the timing of onset as the initial manifestation of lymphoma ("lymphoma-induced") or as triggered by treatment ("HLH during chemotherapy") [5]. Mutations in genes that control the transportation and regulation of granules containing cytotoxic proteins are implicated in cases of familial HLH: Perforin-1 (PRF-1), Unc-homolog-13D (UNC13D), Syntaxin-11 (STX11), Syntaxin binding protein-2 (STXBP2) genes (familial HLH type 2-5, respectively), Ras related protein-27 (RAB27), and the Lyst gene (Chediak-Higashi) [4].

HLH can present with symptoms of fever, jaundice, rash, hepatosplenomegaly, lymphadenopathy, and laboratory abnormalities including hyperferritinemia, pancytopenia, and hypertriglyceridemia. Because of its rarity and symptomatic overlap with other more common conditions, establishing early diagnosis can be challenging [6]. According to HLH-2004 diagnostic criteria, an establishment of a diagnosis requires a mutation in a known causative gene or fulfillment of five out of eight of the following criteria: fever (peak temperature of >38.5°C for >7 days); splenomegaly (spleen palpable >3 cm below costal margin); cytopenia involving >2 cell lines (hemoglobin <9 g/dL [90 g/L], absolute neutrophil count [ANC] <100/mcL [0.10 × 10^9^/L], platelets <100,000/mcL [100 × 10^9^/L]); hypertriglyceridemia (fasting triglycerides >265 mg/dL [3.0 mmol/L] or >3 standard deviations [SD] more than normal value for age) or hypofibrinogenemia (fibrinogen <150 mg/dL [1.5 g/L] or >3 SD less than normal value for age); hemophagocytosis (in biopsy samples of bone marrow, spleen, or lymph nodes); low or absent NK cell activity; serum ferritin >500 ng/mL (>1123.5 pmol/L ng/mL); elevated soluble interleukin-2 (CD25) levels (>2400 U/mL or very high for age) [7].

## Case presentation

A middle-aged male with a 20-year history of alcohol use disorder presented to the emergency department accompanied by his wife for a one-month history of generalized weakness, nonspecific abdominal pain, and loss of appetite. During this time, he experienced occasional episodes of non-bloody, non-bilious vomiting and a single episode of bloody stool. He reported an estimated weight loss of over 20 lb over the past three months. His last drink was four months prior to presentation, but previously consumed 10-20 drinks of liquor daily for 20 years. Social history included marijuana use and occasional cigarette smoking, but no history of intravenous drug use. He works at an elementary school but denies any sick contacts. The patient has not seen a physician in over a decade due to personal preference and came to see a physician now, as persuaded by his partner.

**Figure 1:**
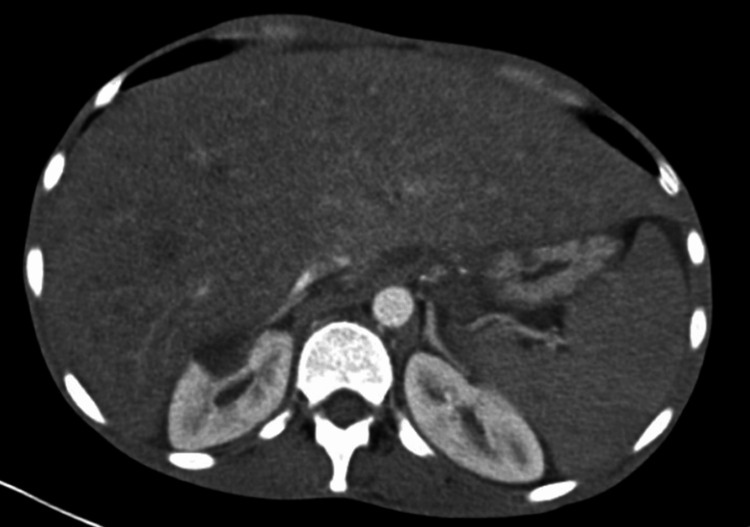
Hepatosplenomegaly

**Figure 2:**
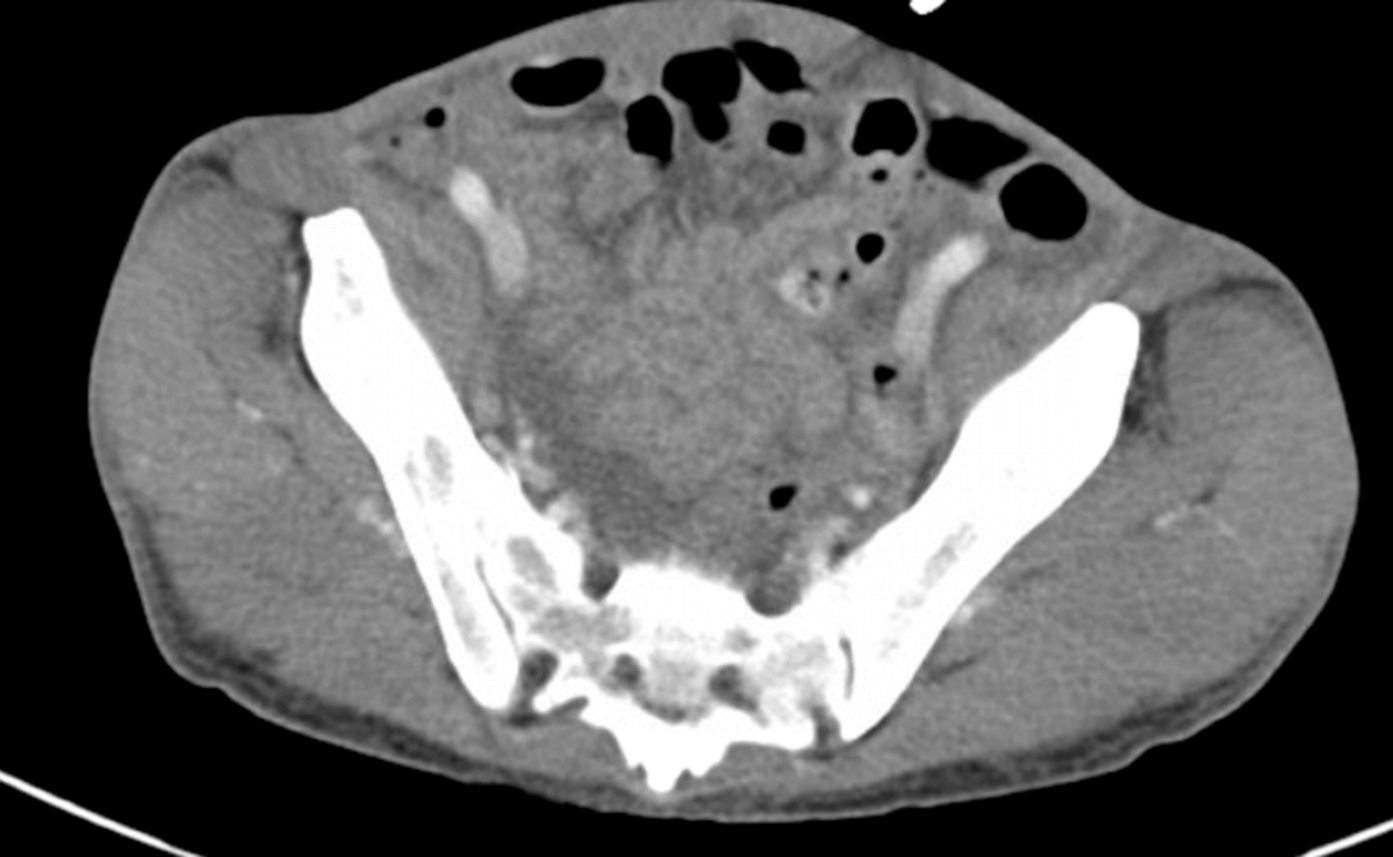
Mild Abdominal-Pelvis Ascites

**Figure 3:**
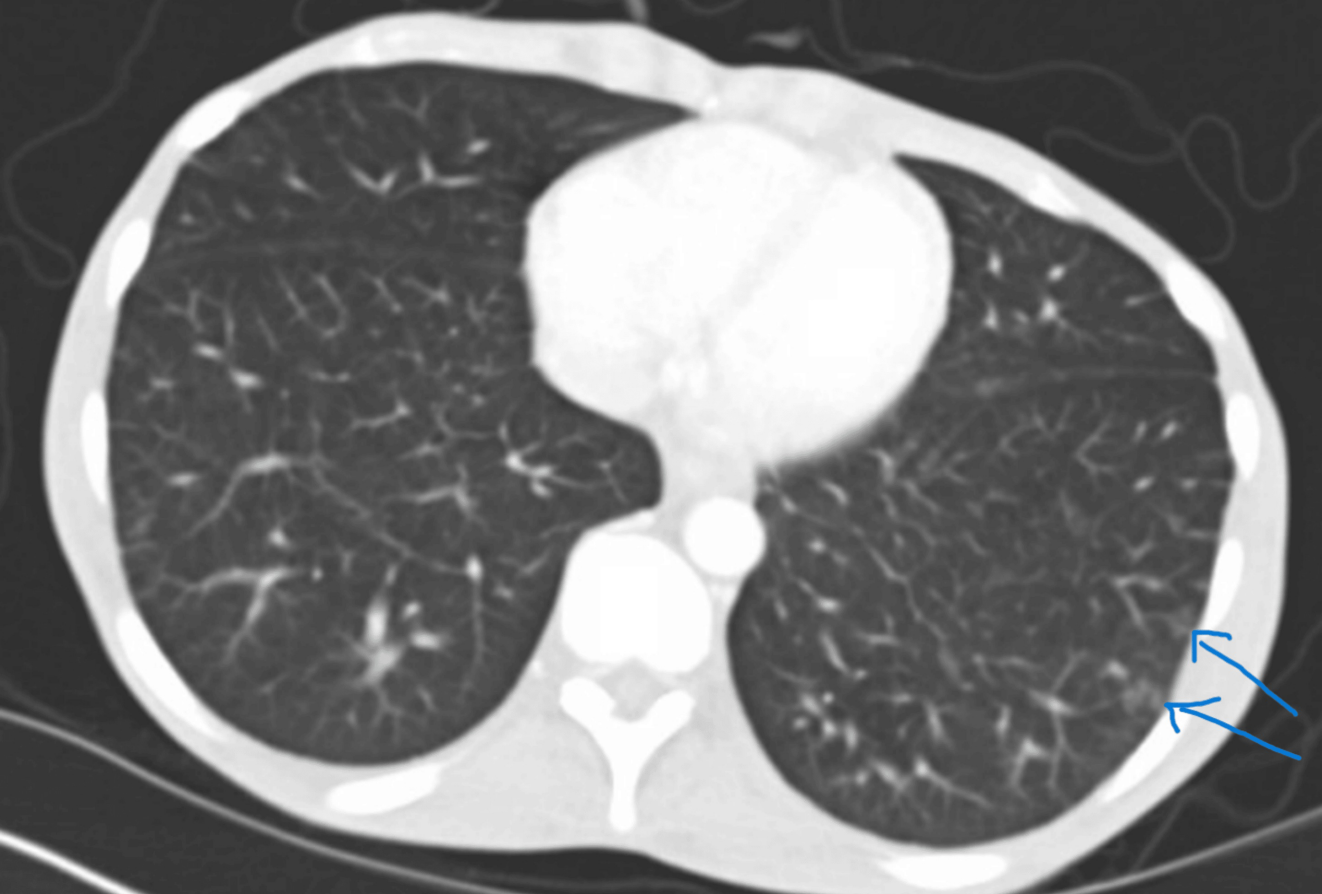
Tree in Bud Opacities Visualized in the Left lung

Physical examination was pertinent for scleral icterus, subcentimeter non-tender firm submental, cervical, submaxillary, and inguinal lymphadenopathy. His abdomen was significant for an enlarged liver span measuring 16 inches and splenomegaly. Aterixis was found bilaterally. Initial laboratory findings were significant for leukopenia with a normal ANC, segmented neutrophilia, bandemia, thrombocytopenia, mild anemia, and hematocrit (Table 1). Given the patient's only mild anemia, these findings were initially not convincing of pancytopenia. The basic metabolic panel revealed hyponatremia (127), hyperkalemia (5.9), hypoglycemia (66), hyperammonemia (55), elevated thyroid-stimulating hormone (TSH) (14.1), elevated lactate (4), and hyperbilirubinemia (8.5). Transaminitis was present with aspartate aminotransferase (AST) (727), alanine aminotransferase (ALT) (189), and alkaline phosphatase (ALP) (738). Albumin was decreased at 2.6. Prothrombin time (PT), international normalized ratio (INR), and activated partial thromboplastin time (aPTT) were all elevated at 28.5, 2.44, and 48.6, respectively. Hepatitis screening panels for A, B, and C, and HIV, were negative. Serum alcohol was less than 3. Computed tomography (CT) of the abdomen and pelvis was significant for hepatosplenomegaly, mild abdominal and pelvic ascites, and multiple tree bud opacities in the left lung (Figures 1-3). He was admitted to the general medical floor for suspected acute liver failure with elevated lactate. Other admitting diagnoses included hypothyroidism and hyponatremia.

**Table 1:** CBC Days 1-3 WBC, white blood cells; ANC, absolute neutrophil count; CBC, complete blood count.

Laboratory Test	Reference Range	Day 1	Day 2	Day 3
WBC (x10^3^/uL)	4.00-10	2.6	2.10	1.3
Hemoglobin (g/dL)	13.2-16	12.2	10.3	8.3
Platelets (x10^3^/uL)	140-440	63	56	49
Neutrophils (%)	40-60	71	73	73
Bands (%)	0-9.0	28	17	6
ANC (x10^3^/uL)	1.8-7.42	2.6	1.9	1.0

Hospital course

During admission, our patient received consultations from hematology, nephrology, and gastroenterology. His pancytopenia became increasingly significant with each consecutive day (see Table 1). Pancytopenia was evaluated to be multifactorial, with the possible etiologies of chronic malnutrition vs liver failure vs disseminated intravascular coagulation (DIC). A fibrinogen level was ordered to evaluate for DIC and found to be low at 23 mg/dL. This required six units of cryoprecipitate for correction. He was also evaluated for myelosuppression secondary to sepsis, supported by findings of leukopenia, hyperthermia of 102°F on day 2 of admission, multiorgan failure, and positive testing for enterovirus and rhinovirus. Initially, he was evaluated with a low suspicion for malignancy as CT imaging showed no masses or lymphadenopathy. Additionally, cancer markers, carcinoembryonic antigen (CEA) and alpha-fetoprotein (AFP), were negative.

Patient's electrolytes were found to be hypoosmolar hyponatremic, explained by poor solute intake, and in addition to chronic alcohol use. Other abnormal electrolyte findings included hypomagnesemia, hypophosphatemia, and hypokalemia suspected to be related to chronic alcohol use and refeeding syndrome.

Inpatient treatment included urea 15 g BID to treat poor solute intake, initiation of 2% hypertonic saline 50 mL/h, and Lasix 20 mg IV. Due to suspected liver failure, he received acetylcysteine for three days for treatment of liver failure. The patient received 10 mg of vitamin K for elevated INR. Esophagogastroduodenoscopy (EGD) was considered; however, it was postponed for concerns regarding hemodynamic stability. He received ceftriaxone 2 g for concern of sepsis from possible spontaneous bacterial peritonitis. Paracentesis was considered and attempted; however, a large enough ascitic pocket was unable to be identified to safely withdraw the fluid. 

On day 2 of admission, ferritin labs were found to be elevated (43,764). The ferritin results stimulated a revaluation of possible malignancy, while simultaneously treating for liver failure and possible superimposed infection. On day 3 of admission, bone marrow aspirate and biopsy were performed. Following the bone marrow biopsy, pulse steroids were started with 1000 mg IV solumedrol for five days, later succeeded by dexamethasone 20 mg IV daily. Throughout the hospital course, considerations were made to transfer the patient to a hospital in which he would be eligible for a liver transplant. The patient also received further workup for the etiology of liver failure, including testing for human herpesvirus 8 (HHV8) (Castleman's), Epstein-Barr virus (EBV), and cytomegalovirus (CMV), which were all found to be negative.

On day 7 of hospitalization, bone marrow biopsy resulted in hypercellular marrow with involvement by large B-cell lymphoma. The bone marrow biopsy showed large, atypical lymphocytes with hemophagocytosis present. Immunohistochemical (IHC) stains showed 30% involvement of atypical lymphocytes, which were positive for CD20, PAX5, BCL6, and MUM1 and negative for CD10, CD30, and CD15. These findings were consistent with lymphoma and concurrent hemophagocytosis. On day 9 of hospitalization, a core biopsy of the right axilla was performed, in which pathology showed lymphoid tissue consisting of a polymorphous population of small to medium lymphocytes and an admixture of histiocytes. The IHC staining of the lymphoid tissue was positive for CD20, PAX5, CD3, and CD5 and negative for BCL1, BCL6, and BCL10. MUM1 highlights scattered plasma cells and rare immunoblasts. Ki-67 was approximately 30%. However, there was no diagnostic evidence of lymphoma involvement due to the limited sample.

The patient was transferred out on day 10 to an alternative institution for initiation of cancer treatment. The patient was ultimately diagnosed with Stage IV diffuse large B-cell lymphoma (DLBCL) and secondary HLH due to DLBCL. The patient received urgent inpatient chemotherapy. He was treated with five days of cyclophosphamide followed by inpatient therapy of “DA-R-EPOCH” drug therapy, which includes dose-adjusted rituximab, etoposide, prednisolone, vincristine, cyclophosphamide, and doxorubicin. At the time of drafting this case report, the patient had successfully completed his second round of inpatient chemotherapy.

## Discussion

What makes this case so interesting is how the patient's presentation and findings could have been plausibly explained by diseases other than HLH, such as chronic liver failure, alcoholic hepatitis, or even hemochromatosis. This ambiguity is compounded by the lack of medical care over the past decade. It was unclear to the providing care teams if the patient had an undiagnosed chronic liver disease in the time leading up to his presentation. Timing is key in diagnosing HLH, so it is important to be familiar with the nuances between HLH and alternative diagnoses to result in a timely diagnosis.

In a retrospective study [8] conducted at Beijing You-An Hospital, Beijing, China, on 11 patients with HLH who were initially diagnosed with liver failure, it was found that the average period from initial diagnosis of liver failure until correct diagnosis of HLH was 17 days. This study further illustrates the point of how HLH can mask itself with a liver failure presentation and ultimately delay the correct diagnosis of an underlying malignancy. Similar to our patient, this study had plausible explanations for the patients with liver failure other than HLH, i.e., alcohol use, HBV, CMV, and septic shock. Given the ambiguity and high mortality of HLH when it presents with liver failure, this article raises an important question: which individuals with liver disease should be screened for HLH, considering that the presentation is the same?

This patient's social history, physical examination, and laboratory findings were consistent with chronic liver disease, specifically, alcoholic hepatitis. He presented with hepatomegaly, AST-dominant transaminitis, elevated bilirubin, and hyponatremia, all complications of chronic liver disease. There are multiple etiologies of hematological abnormalities in individuals with liver disease, and it can present in up to 75% of liver disease patients [9]. Hepatitis with associated aplastic anemia is a rare condition that can present with hematological abnormalities, including anemia, neutropenia, thrombocytopenia, and significantly decreased reticulocyte count [10]. Pancytopenia can also be seen in patients with liver failure due to chronic alcohol use. Many individuals with alcohol use disorder tend to be malnourished, with B12 and/or folate deficiency. Additionally, as the liver function declines, so does the synthesis of clotting factors, leading to thrombocytopenia and risk for small gastrointestinal bleeds, which can worsen existing anemia. Alcohol itself is a toxin to the bone marrow, and chronic use can suppress the function [11]. Although leukocytosis can be seen in alcoholic liver disease, leukopenia, as seen in our patient, can be found. In a prospective study conducted by Sharma University of Health Sciences [12] on patients with alcoholic liver cirrhosis, it was found that patients with higher Model for End-Stage Liver Disease (MELD) scores tend to have leukocytopenia in comparison to those with lower MELD scores. In patients with MELD scores above 30, leukopenia is more commonly seen. In patients with MELD scores above 40, only leukopenia was seen [12]. Previous studies on the range of transaminitis reveal that alcohol hepatitis generally has AST and ALT below 300 and typically does not exceed values of 500 [13]. When transaminases are elevated above 500, this should allow clinicians to widen their differential diagnosis.

While the elevated ferritin levels in our patient were the ultimate evidence of HLH, this is not always the case. In pediatrics, hyperferritinemia is highly specific for HLH, with an estimated specificity of 98% for HLH [14]. In adults, hyperferritinemia is not limited to HLH. A study [15] performed at Kyushu University Hospital, Japan, included 118 patients with hepatitis, defined by ALT over 10× the normal limit, and found that individuals with alcohol hepatitis can have astonishingly elevated serum ferritin. In patients with acute liver failure, serum ferritin levels were found to range from 45 to 876,790 ng/mL. It is important to note that the maximum serum ferritin levels in patients with acute hepatitis, excluding those with acute liver failure, did not exceed 32,000 ng/mL. An additional consideration about this study is that only nine of the patients had serum ferritins that exceeded 50,000 ng/mL. This study is fortified by the fact that participants who met the criteria for HLH were excluded, which avoids a confounding factor for the increase in serum ferritin [15]. Given that elevated ferritin can be seen in hepatitis and liver failure, it added ambiguity to our case as an additional support to a liver pathophysiology in lieu of HLH.

Hemochromatosis, another plausible diagnosis for our patient, also complicates the ambiguity of this case. Hemochromatosis can present with hepatomegaly, transaminitis, and liver disease, progressing more rapidly in younger patients who consume excess alcohol. Iron deposition occurs insidiously, resulting in clinical manifestations after age 40 in men and postmenopausal women. Our patient had findings supportive of hemochromatosis, including liver function tests three times the upper limit of normal, a ferritin level above 43,000, transferrin saturation of 89%, subclinical hypothyroidism, and hyponatremia. Another finding that supports the diagnosis of hemochromatosis includes skin discoloration. Skin discoloration was not observed during the physical examination, but further conversation with the patient's wife revealed color changes. In individuals with darker skin tones, skin manifestations of conditions like jaundice or melanoma can be subtle or appear in less commonly examined areas, such as the palms, soles, and oral mucous membranes [16,17]. The patient was tested for hemochromatosis common mutations, with negative results. However, these results were not available during the initial hospitalization.

On physical examination, the patient was found to be cachexic with firm palpable axilla and inguinal lymphadenopathy found by multiple providers on different teams providing treatment. The CT abdomen and pelvis did not report any findings of lymphadenopathy. This highlights the critical role of a thorough physical examination. Without it, the patient might have been misdiagnosed with liver failure, missing the opportunity for a bone marrow biopsy to detect malignancy.

## Conclusions

This report’s focus is to publicize the ambiguity of HLH with a liver presentation from other possible diagnoses that could explain liver failure, including hemochromatosis, chronic alcohol use, and alcoholic hepatitis. Key distinctions that can help differentiate HLH from alcoholic hepatitis include both the level and the regularity of transaminitis and hyperferritinemia. Transaminitis in alcoholic hepatitis is typically under 300 units/L, and if values exceed 500 units/L, clinicians should widen their differential diagnosis. Hyperferritinemia can also be seen in alcoholic hepatitis; however, values exceeding 32,000 are rarely seen unless presenting with concomitant acute liver failure. In the early stages, alcohol hepatitis will commonly present with leukocytosis and, more specifically, neutrophilia. When the MELD score is above 30, leukopenia will predominate. Many of the diagnostic criteria for HLH, including splenomegaly, hypofibrinogenemia, bi-cytopenia, and hyperferritinemia, are often already present in patients with chronic liver disease. Currently, there are no guidelines to aid practitioners in deciding when to pursue HLH testing (i.e., biopsy or NK testing) in populations with chronic liver diseases. We hope this case report promotes further research in the field to explore additional key distinguishing factors that can help differentiate when HLH should be suspected in patients with liver disease.
